# Pandora's Box

**DOI:** 10.1192/bji.2023.38

**Published:** 2024-02

**Authors:** Eleni Palazidou

## ‘Doctors pour drugs of which they know little, to cure diseases of which they know less, into patients of whom they know nothing’ (Voltaire)

We have come a long way since Voltaire's times, but how safe are we in drug prescribing and can we do better? Over a hundred years ago, Sir William Osler cautioned ‘*the physician without physiology and chemistry flounders along in an aimless fashion, never able to gain any accurate conception of disease, practising a sort of popgun pharmacy, hitting now the malady and again the patient, he himself not knowing which’.*

Today we are bombarded with an ever-increasing volume of drugs that we can prescribe. Many conditions previously deemed not treatable can now be cured or controlled by drugs, and our patients can enjoy a better quality of life. However, drug treatments have the potential to cause serious harm and death. According to the World Health Organization (WHO), ‘Unintended, harmful reactions to medicines (known as adverse drug reactions) are among the leading causes of death in many countries’. Commonly, patients are prescribed more than one drug to manage resistant-to-treatment disorders or multiple comorbidities, physical and mental. Polypharmacy, often used as a critical term, is a reality to today's prescribing doctor and, as Sir William Osler said, ‘The true polypharmacy is the skilful combination of remedies’. There are numerous pharmacokinetic, pharmacodynamic and idiosyncratic reactions to consider when prescribing, in addition to taking care to avoid any errors.

In recent years, the role of drug prescribing supervision has been delegated to pharmacists in many countries. Although this is very useful, in that prescriptions are scrutinised by these professionals in hospital and community pharmacies and errors can be picked up, is it enough? The pharmacists know about the drugs, but they don't have the expertise of the prescribing doctor in human physiology and pathophysiology. The ultimate responsibility remains with the doctor.

The UK General Medical Council, after a finding that one in ten hospital prescriptions contained errors, commissioned the Prescribing Safety Assessment (PSA). The PSA assesses the skills, judgement and knowledge required to prescribe and supervise the use of medicines. It is voluntarily adopted by all medical schools in the UK and has been running for the past 10 years. Since 2016, Foundation Year 1 doctors have been required to pass the assessment before entering Year 2.

It is planned that next year (2024), a National Medical Licensing Assessment will be introduced in the UK to standardise medical schools’ final exams from the year 2025 and replace the Professional and Linguistic Assessments Board for international medical graduates. This prompted the commissioning of an independent review of the PSA, the Dacre Review, by the Joint Medical Schools Council and the British Pharmacological Society, with a view to considering its place in future undergraduate and postgraduate education, as well as licensing. The review examined the impact and usefulness of the PSA, using the longitudinal performance of candidates and schools, stakeholder feedback and surrogate markers of prescribing safety in UK healthcare practice. It concluded that the PSA provides a reliable measure of prescribing competence measured against the national standard. They found most candidates achieved this standard at first attempt. The review recommends the standardisation of the PSA delivery nationally and suggest that for it to be made a requirement prior to clinical practice is pragmatic and can sustainably prioritise patient safety.

Interestingly, the review found newly qualified doctors to be safer in prescribing then older doctors. Perhaps we should all be required to have refresher courses in patient safety as a requirement for our continuing professional development and continuing license to practise?

Magavern EF, Hitchings A, Bollington L, Wilson K, Hepburn D, Westacott RJ, et al UK Prescribing Safety Assessment (PSA): the development, implementation and outcomes of a national online prescribing assessment. Br J Clin Pharmacol [Epub ahead of print] 4 Oct 2023. Available from: 10.1111/bcp.1591937793701.

## Plasticisers and solvents and offspring IQ

The more we explore the impact of pollutants on our health and on the flora and fauna around us, the more we come to realise the damage caused. There is increasing concern about plasticisers and solvents in products we use in everyday life and their potential for neurotoxicity. Phthalates are commonly found in many commercial products such as personal care items, cosmetics and food packaging. A study from the NYU Grossman School of Medicine in collaboration with the Barcelona Institute for Global Health examined the effect of phthalates, which are contained in such products, on the brains of babies born to mothers exposed to these substances during pregnancy. They recruited 775 mother–child pairs from Generation R, a paediatric neuroimaging cohort in Rotterdam, The Netherlands. Phthalate exposure in mothers was assessed by testing for the presence of pthalate metabolites monoethyl phthalate (mEP) and monoisobutyl phthalic acid (mIBP) in urine samples obtained during pregnancy. mEP is a metabolite of diethyl phthalate, which is used to render plastics more flexible and is contained in cosmetic products. Brain volumetric studies were carried out in children by means of magnetic resonance imaging scans at age 10, and their IQ was assessed at age 14.

The researchers found that higher urine concentrations of these phthalate metabolites during pregnancy were associated with a reduction in grey matter in the offspring at age 10 and lower IQ at 14, the latter possibly related to the lower grey matter volume. They also found that prenatal exposure to mIBP was associated with smaller white matter volume in the brains of girls.

The authors caution that lax regulations and the widespread use of phthalate-containing products present a significant public health concern.

Ghassabian A, van de Dries M, Trasande L, Lamballais S, Spaan S, Martinez-Moral, MP, et al Prenatal exposure to common plasticizers: a longitudinal study on phthalates, brain volumetric measures and IQ in youth. Mol Psychiatry [Epub ahead of print] 29 Aug 2023. Available from: 10.1038/s41380-023-02225-6PMC1106244737644173.

## Of body and mind

Physical health disparities are observed and persist globally in both low- and high-income countries. It is well known that people with severe mental illness often suffer from physical health problems, and these are frequently due to more than one physical condition. It is also known that mental and/or physical multimorbidity has serious adverse effects on people's illness outcomes, day-to-day functioning and general well-being, as well as shortening their lifespan.

The authors of a recent meta-analysis and systematic review assessed the strength of the association between mental illness and physical multimorbidity. In a literature search, they identified 19 studies with a total of close to 200 000 patients with severe mental illness (schizophrenia and other psychotic disorders, bipolar disorder and psychotic depression) and found that they were twice as likely to experience chronic physical conditions as those without mental illness.

Based on their findings, the authors stress the urgent need for a ‘multidisciplinary approach for those with severe mental illness and physical multimorbidity’ in order to improve physical, mental and social outcomes.

Pizzol D, Trott M, Butler L, Barnett Y, Ford T, Neufeld SA, et al Relationship between severe mental illness and physical multimorbidity: a meta-analysis and call for action. BMJ Ment Health 2023; 26(1): e30087010.1136/bmjment-2023-300870PMC1061903937907331.

## Don't forget your coffee!

Pandora, a self-confessed coffee afficionado, has repeatedly exalted the benefits of coffee drinking to health, based of course on evidence. May I remind the readers that previous Pandora's Box articles have reported on several publications that demonstrated the value of moderate coffee drinking with respect to human health. It protects the liver from alcohol damage (not to be taken as license to drink!), as well as having other health benefits and being associated with a longer lifespan. Now let's find out how it achieves these benefits.

A new study explored possible mechanisms via which coffee may be beneficial to our health. The authors investigated the effects of trigonelline, an alkaloid contained in coffee, on senescent mice's memory and spatial learning, as well as performing a variety of molecular biology tests. After a month-long administration of trigonelline, the mice exhibited improved cognitive ability on various tasks. The researchers also obtained a whole-genome transcriptome of the mouse hippocampus. They found that a number of biological processes, which included nervous system development, mitochondrial function, adenosine triphosphate synthesis, and signalling pathways related to inflammation and neurotransmitter release, functioned significantly better in the treated mice compared with the untreated controls. They found, in addition, that trigonelline decreased levels of pro-inflammatory cytokines (TNF-α and IL6) and increased those of neurotransmitters (dopamine, noradrenaline and serotonin) in the hippocampus.

In summary, trigonelline improves brain function, at least in the hippocampus of mice, and the authors suggest that it may have the potential to improve memory in humans in older age.

Aktar S, Ferdousi F, Kondo S, Kagawa T, Isoda H. Transcriptomics and biochemical evidence of trigonelline ameliorating learning and memory decline in the senescence-accelerated mouse prone 8 (SAMP8) model by suppressing proinflammatory cytokines and elevating neurotransmitter release. Geroscience [Epub ahead of print] 18 Sep 2023. Available from: 10.1007/s11357-023-00919-xPMC1082827037721682.

## The Rabat Declaration

Conflict, persecution, oppression and lack of opportunity in various parts of the world drive people out of their home countries to migrate to safer places. According to the WHO, one in eight people in the world is either a migrant or is forcibly displaced. Not all host countries are welcoming, and the Covid-19 pandemic has highlighted healthcare discrepancies.

The Third Global Consultation on the Health of Refugees and Migrants took place in the Kingdom of Morocco, co-hosted by the WHO, the UN Migration Agency, UNHCR and the UN Refugee Agency, and was attended by 50 United Nations Member States. The meeting ended with the Rabat Declaration. The countries that supported the declaration made a commitment to:

- accelerate efforts to improve the health of refugees, migrants and their host communities;
- address the root causes that negatively influence their health; and
- work towards including health and social protection considerations in national policies related to refugees and migrants.

They also re affirmed the right of every human being, including refugees and migrants to enjoy the highest attainable standard of physical and mental health. The countries that supported the declaration committed to including refugee and migrant populations and their host communities in policies and plans for prevention, preparedness, response and recovery with respect to pandemics and other public health emergencies, while strengthening international and cross-border collaboration. They also pledged to foster inclusive financing mechanisms to reduce budget pressures on national systems and promote the meaningful participation of refugees and migrants in health policy discussions to identify and design appropriate interventions for their health needs.

*Rabat Declaration (English version)*. Third Global Consultation on the Health of Refugees and Migrants, 13 June 2023

